# Global and Regional Patterns in Noncommunicable Diseases and Dietary Factors across National Income Levels

**DOI:** 10.3390/nu13103595

**Published:** 2021-10-14

**Authors:** Sooyoung Kang, Minji Kang, Hyunjung Lim

**Affiliations:** 1 Department of Medical Nutrition, Graduate School of East-West Medical Science, Kyung Hee University, Yong-in 17104, Korea; ksyblue1127@naver.com (S.K.); mingi4182@gmail.com (M.K.); 2 Research Institute of Medical Nutrition Nutrition, Kyung Hee University, Seoul 02447, Korea

**Keywords:** noncommunicable diseases (NCDs), national income levels, deaths, dietary factors, metabolic risk factors

## Abstract

Background: Noncommunicable diseases (NCDs) are the leading global cause of death and share common risk factors. Little quantitative data are available on the patterns of each NCDs death and dietary factors by national income level and region. We aimed to identify the trend of NCDs deaths and dietary factors with other health-related behaviors across national income levels and geographical regions. Methods: Three databases were collected, including the World Health Organization, Food and Agriculture Organization, and World Bank in 2014. These were analyzed to describe the trend for NCDs deaths and dietary factors with health-related behaviors across national income levels (high income, upper-middle income, lower-middle income, and low income) from 151 countries using variance-weighted least-squares linear regression. Results: Lower-middle-income and low-income countries in Africa and Asia had higher death rates of NCDs. More than 30% of the population had raised blood pressure with higher carbohydrate intake and lower protein and fat intake compared to high-income European countries in 2014. High-income countries had the highest prevalence of raised total cholesterol, overweight, and obesity, the highest total energy, fat, and protein intake, and the highest supplies of animal fat, stimulants, sugar and sweetener, vegetable oil, and milk, as well as insufficient activity with an increasing trend (*p* for trend < 0.001). Conclusion: There were differences in NCDs risk factors and dietary factors by national income and region. Accordingly, measures should be taken to suit the situation in each country. Our findings have significance for health workers and health policies preventing and controlling the rise of NCDs.

## 1. Introduction

Noncommunicable diseases (NCDs), including cardiovascular diseases (CVDs), cancers, diabetes, and respiratory diseases, cause around two-thirds of the global deaths [1], which were specified as one of the major challenges for sustainable development in the Global Sustainable Development Report [2]. Total deaths from NCDs accounted for 70% (41 million) of 57 million deaths worldwide in 2016 [3]. On the national level, the threat of NCDs also goes with a significant loss of productivity caused by the inability to work and absenteeism, which ultimately leads to a decline in national income [4]. 

Death rates from NCDs vary depending on the level of the national economy [4]. In low-income countries (LICs), 43% of deaths (aged 30~69 years) occur due to NCDs, while high-income countries (HICs) have only 25% of deaths caused by NCDs [3]. However, low-income and lower-middle-income countries (LLMICs) do not have sufficient resources and capacity to settle these NCDs [5]. NCDs have become a more serious issue in LLMICs because of the most considerable burden of morbidity and mortality [4]. Thus, the costs for NCDs are becoming a growing burden, which affects the quality of life and puts more pressure on the health system in LLMICs [6]. In contrast, HICs have regional differences in their NCDs mortality. Therefore, efforts have been made to understand this pattern of wide variation with risk factors [7]. 

The development of global and regional NCDs mortality and dietary factor profile across national income levels provides key information required for planning prevention and controlling activities for health workers and health policies. A reliable analysis of NCDs mortality and dietary factors is especially important for preventing or modifying disease. Until recently, the global distribution of NCDs mortality and dietary factors has been well established, but little evidence for the comprehensive distribution of NCDs mortality and dietary factors by a trend of national income levels has been published with a limited number of countries [8]. 

To better understand the global and regional pattern for NCDs mortality and dietary factors according to national income level, we described the trend of NCDs mortality and dietary factors for NCDs across national levels using data from the WHO and methods from a geographic information system (GIS) by visualization, which has been designed to manage all types of spatial or geographical data and is helpful to identify patterns in the data [9]. 

## 2. Subjects and Methods

### 2.1. Study Population

**Figure 1:**
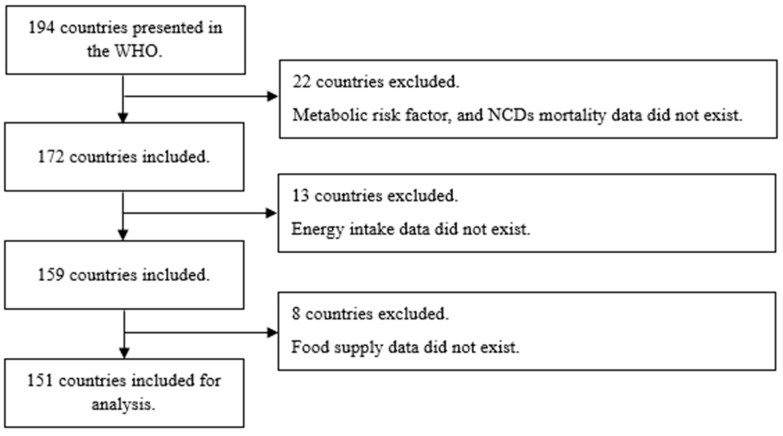
The flow diagram of selection between countries.

The study population was based on a census of each country selected based on the World Health Organization (WHO) [10]. Forty-three countries were excluded due to the absence of data: 22 countries for a lack of data on the number of deaths from all kinds of NCDs per 100,000, 13 countries for the absence of total energy intake (kcal/day/capita) data, and 8 countries for the absence of supplies of several food items (kcal/day/capita); eventually, a total of 151 countries were selected (*n* = 6,979,376,821) (Figure 1). The standard year of this population was 2014.

### 2.2. Classifications

Countries were divided into regions, continents, and the direction in which they were located. It can be classified into five continents: Africa, America, Asia, Europe, and Oceania.

Socioeconomics data, including gross national income (GNI) and income levels, were gathered from the World Bank [11]. Income levels were categorized based on the World Bank Analytical Classifications: levels 1, 2, 3, and 4 correspond to high-income (39 countries, GNI per capita over USD 12,615), upper-middle-income (43 countries, GNI per capita; USD 4036–12,475), lower-middle-income (38 countries, GNI per capita; USD 1026–4035), and low-income (31 countries, GNI per capita under USD 1025) levels, respectively (2014). GNI per capita was derived from the Atlas method by the World Bank. The currency of GNI per capita is currently USD (2014).

### 2.3. Data Collection

#### 2.3.1. NCDs Mortality, Metabolic Risk Factors, and Health-Related Factors

The data on mortality of NCDs, metabolic risk factors, and health-related behavior were obtained from the WHO [12]. 

Age-standardized comparable estimates were conducted to compare indicators between countries [13], and the standard age of all populations was over 18. All age- and gender-specific mortality rates were estimated from the amended life table published by the World Health Statistics in 2014 [14]. The WHO standard population was used to calculate age-standardized death rates of CVDs, diabetes, cancers, and chronic respiratory diseases [15]. Proportional mortality for CVDs, diabetes, cancers, and chronic respiratory diseases were reported for 2012 [13,16]. The risk of premature death from target NCDs (%), total NCDs deaths, NCDs deaths under the age of 70 (%), all NCDs (deaths per 100,000), and deaths of cancers, cardiovascular diseases, diabetes, and chronic respiratory diseases per 100,000 were selected as data of mortality for NCDs. 

The estimated metabolic risk factors, body mass index (BMI), blood glucose level, blood cholesterol, and blood pressure were defined as below [17]: 

Diabetes (Raised fasting blood glucose): Fasting blood glucose ≥ 7.0 mmol/L or on medication;

Hypercholesterolemia (Raised total cholesterol): Total cholesterol ≥ 6.2 mmol/L;

Overweight: Body mass index (kg/m^2^) ≥ 25, Obesity: Body mass index (kg/m^2^) ≥ 30;

Hypertension (Raised blood pressure): Systolic blood pressure over 140 mmHg or diastolic blood pressure over 90 mmHg.

Health-related behaviors included insufficient physical activity, smoking, and drinking alcohol. Following the WHO standard, physical activity was defined as activities that can occur throughout daily life (e.g., during the time for work/household, transport, and leisure activities). Smoking was defined as consuming smoked tobacco products or smokeless tobacco. Alcohol consumption was categorized as nondrinkers and drinkers.

#### 2.3.2. Food Supply

The data of food supply (production + imports − exports + changes) and energy intake was derived from the Food and Agriculture Organization (FAO) [18]. The data from the FAO included the following: total energy intake (kcal/capita/day), fat supply (kcal/capita/day), protein supply (kcal/capita/day), fruit, vegetable, cereal, meat, animal fat, vegetable oil, milk, stimulants, and sugar and sweetener supplies (kcal/capita/day).

### 2.4. Statistical Analyses

The data were weighted by the total population size. To investigate the relationship between dietary factors and NCDs by income level, the trends between factors were assessed by variance-weighted least-squares linear regression analysis because the income level is a categorical variable, and the weight was required to analyze trends. All data were expressed as means and standard deviations (SD). Stata 13.0 was used (StataCorp LP, College Station, TX, USA).

To visualize spatial relationships, ArcGIS was used (ArcGIS 10.4.1, Esri, Berkeley, CA, USA). Several factors were selected for expression on the maps. The categorical variables, including income level, were presented as the gradation of colors for each country on most maps.

## 3. Results

Globally, NCDs and NCDs mortality had trends across national income levels in 2014. The prevalence of metabolic risk factors and mortality rates of NCDs by income level are shown in Table 1. There were clear increasing trends of raised blood pressure (HICs: 18.9% vs. LICs: 29.7%), all NCDs deaths, risk of premature death, NCDs deaths under the age of 70, and death from CVDs, diabetes, and chronic respiratory disease with the decrease in income level (*p* for trend < 0.001). NCDs deaths and raised blood pressure were lowest in HICs. In contrast, obvious increasing trends in raised fasting blood glucose, total cholesterol, overweight, obesity, and death from cancers were observed with the increase in income level (*p* for trend < 0.001).

**Figure 2:**
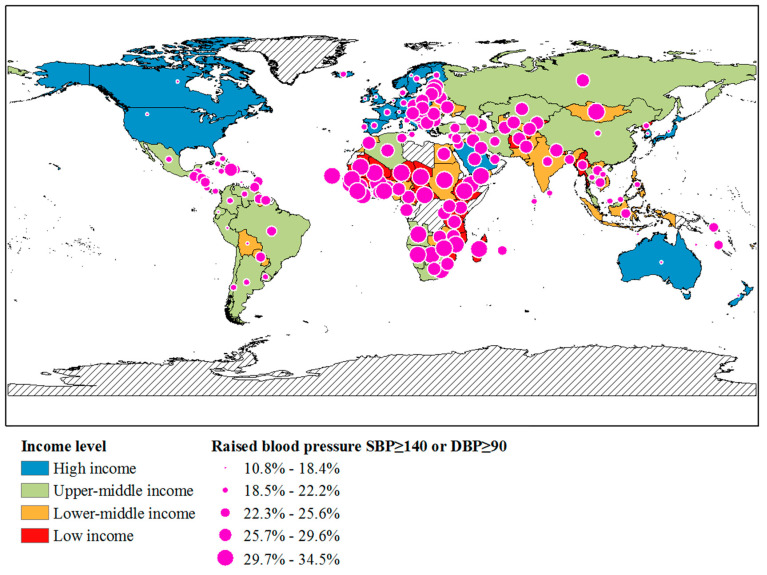
Worldwide distribution of income level and raised blood pressure, Systolic blood pressure (SBP) ≥ 140 or Diastolic blood pressure (DBP) ≥ 90 (%, age standardized, both genders).

The national income level was lower, and the percentage of populations with raised blood pressure was higher (Figure 2). Most obviously, about 30% of the population of eastern, western, middle, and southern Africa and Mongolia in Asia with LLIMCs had raised blood pressure, but other countries had a lower population of raised blood pressure.

**Figure 3:**
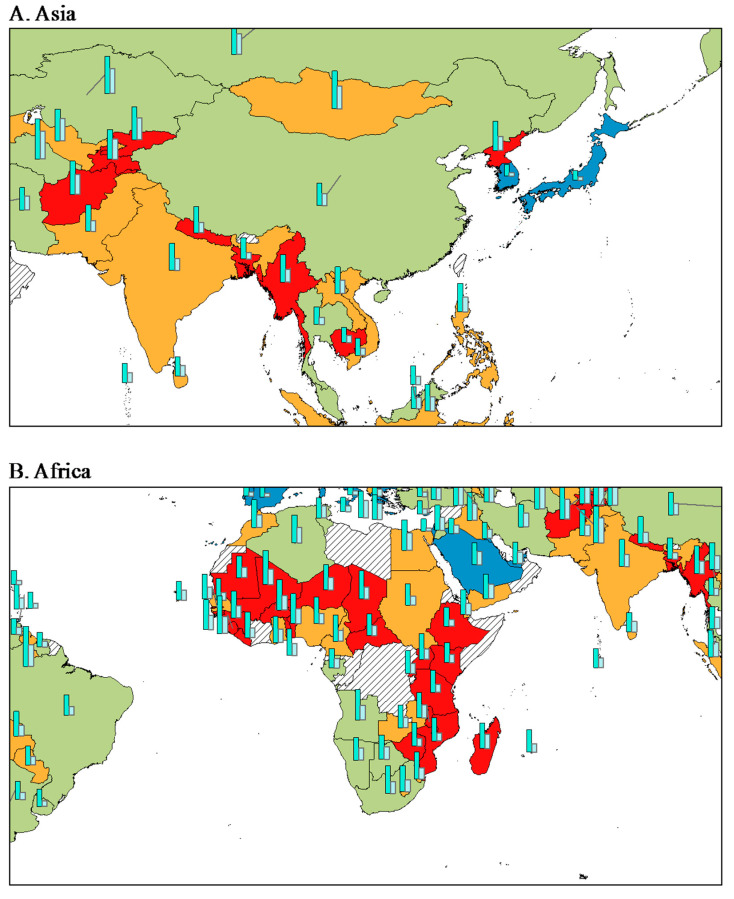

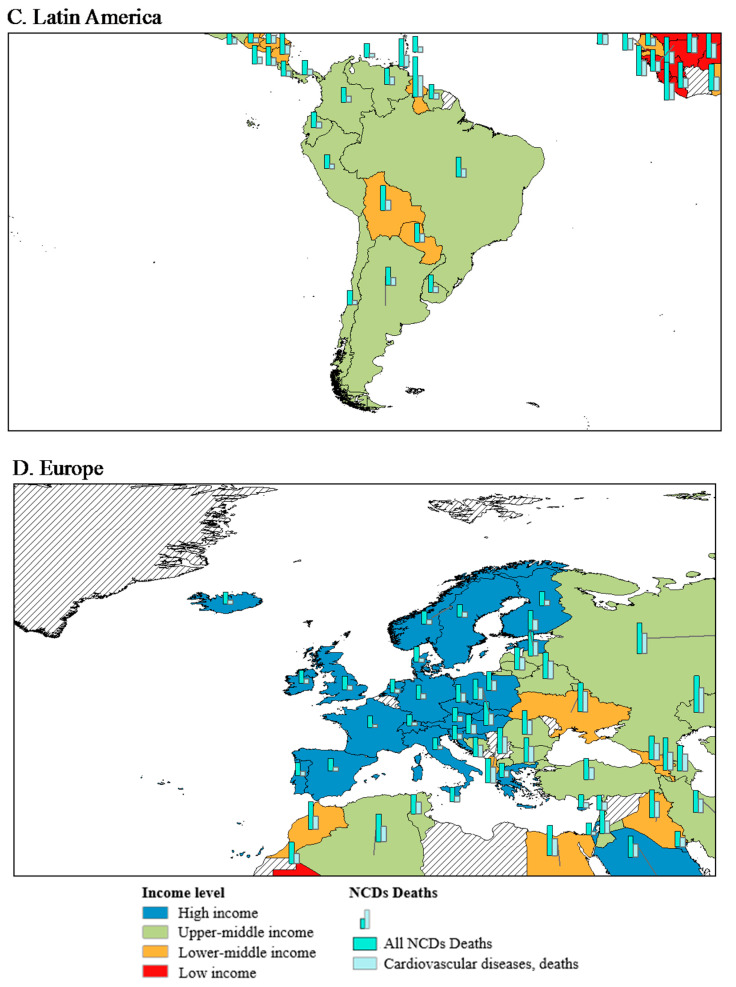
Distribution of income level and NCD deaths (all NCDs and CVDs, per 100,000).

From a geological perspective, countries of similar income levels are concentrated in specific regions (Figure 3). In Asia (Figure 3A) and Africa (Figure 3B), all deaths from NCDs and CVDs in HICs (e.g., South Korea and Japan) were lower than those of LICs (e.g., Afghanistan, Tajikistan, Kyrgyzstan, Ethiopia, Kenya, Madagascar, Mali, and Burkina Faso). Similarly, in Latin America (Figure 3C) and Europe (Figure 3D), all deaths from NCDs and CVDs of Bolivia, Paraguay, and Ukraine, which are LMICs, were higher than those of HICs in Europe.

**Figure 4:**
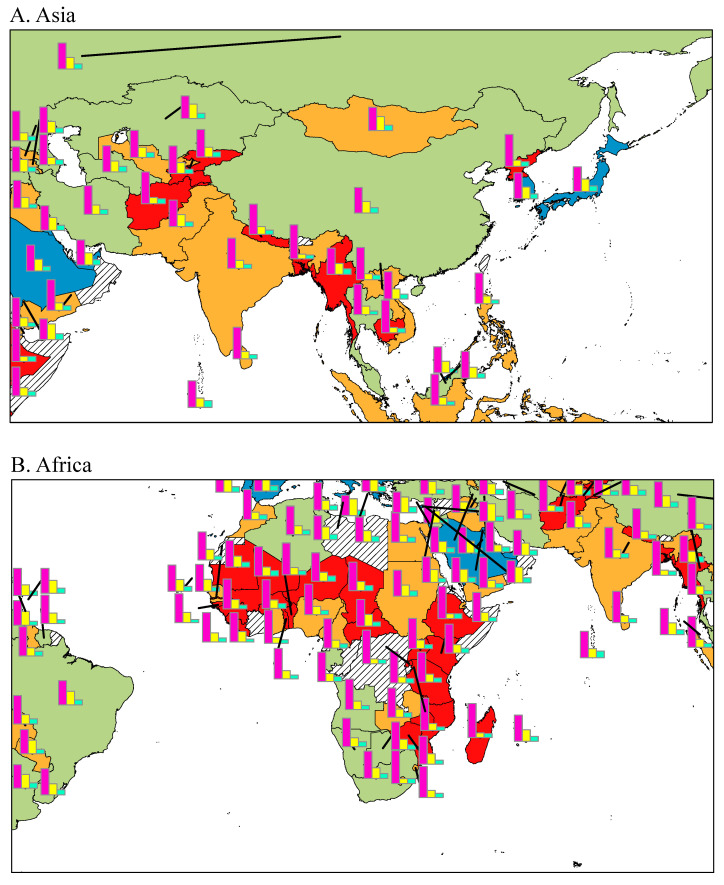

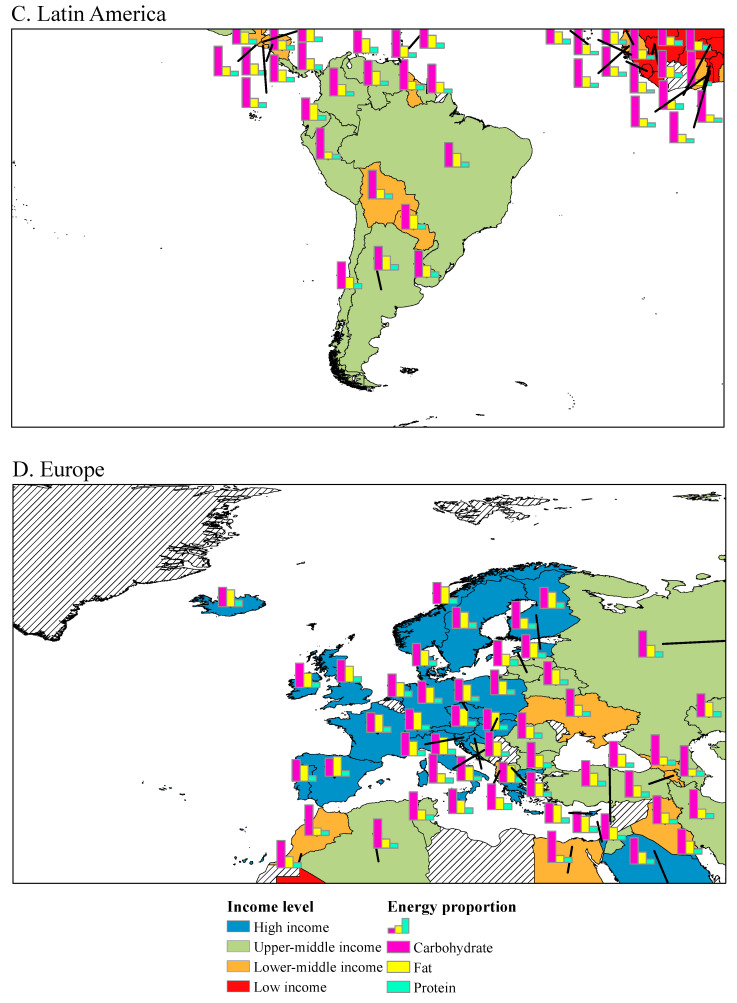
Energy proportion of carbohydrates, protein, and fat for each country (%).

Nutrition composition, food supply, and health-related behaviors according to the income level of the countries are shown in Table 2. We see increasing trends in the intake of total energy (HICs: 3355 ± 287 kcal vs. LICs: 2274 ± 210 kcal), fat (HICs: 35.4 ± 5.7% vs. LICs: 16.7 ± 4.2%), and protein (HICs: 12.1 ± 0.7% vs. LICs: 10.5 ± 1.3%) with increasing income level (*p* < 0.001). In contrast, there is evidence of increasing carbohydrate intake (HICs: 52.5 ± 5.8% vs. LICs: 72.8 ± 4.0%) with decreasing income level (*p* for trend < 0.05). As in the region, carbohydrate intake in Africa was higher, by the same in LLIMCs in Asia, Latin America, and Europe, whereas fat and protein intake were lower than those in HICs and upper-middle-income countries (Figure 4). On the contrary, HICs in Europe and Asia had a higher intake of protein and fat, whereas they had a lower intake of carbohydrates.

Regarding foods, there are increasing trends in most food supplies such as fruit, vegetable, meat, animal fat, vegetable oil, stimulants, and sugar and sweetener with income level, although there is evidence of decreasing cereal supply with income level (*p* < 0.001) (Table 2). Among health-related behaviors, similar trends are observed in smoking (HICs: 32.1 ± 12.8% vs. LICs: 23.5 ± 11.9%) and insufficient activity (HICs: 28.2 ± 10.1% vs. LICs: 14.4 ± 6.9%) with increasing income level (*p* for trend < 0.05), whereas there is an increasing trend with alcohol consumers with decreasing income level (HICs: 38.5 ± 27.8% vs. LICs: 41.0 ± 23.6%; *p* for trend < 0.05).

**Figure 5:**
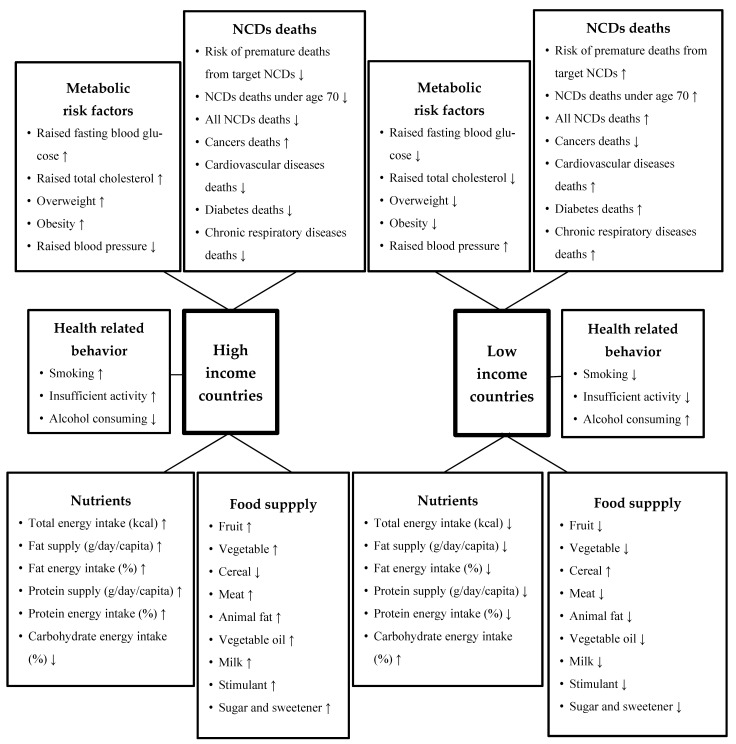
Relative comparison of high- and low-income countries in metabolic risk factors, mortality, and health-related behavior, energy intake, and food supply.

A summary for comparing NCDs mortality and risk factors, including dietary factors in HICs and LICs, is shown in Figure 5. In HICs, the prevalence of raised total cholesterol, overweight, and obesity is highest. In contrast, the risk of premature deaths from target NCDs, NCDs deaths under the age of 70, all NCDs deaths, CVDs, diabetes deaths, and chronic respiratory diseases deaths are lowest. In the aspects of dietary factors, total energy, fat, and protein intake, and supplies for animal fat, stimulants, sugar and sweetener, vegetable oil, and milk were highest, whereas the carbohydrate and cereal intake were lowest in HICs. Concerning health-related behaviors, insufficient activity was highest in HICs.

On the other hand, LICs tend to have the lowest raised fasting blood glucose, total cholesterol, and overweight/obesity, whereas raised blood pressure, the number of NCDs deaths under the age of 70, and diabetes deaths were highest. The total energy intake, fat intake, and protein intake were lowest, whereas these countries have the highest carbohydrate and alcohol consumption, and vegetable, vegetable oil, milk, meat, animal fat, stimulants, and sugar and sweetener supplies were lowest. Insufficient activity and smoking were also lowest in LICs.

## 4. Discussion

This study identified broad trends in global and regional NCDs mortality and risk factors and dietary factors based on national income level. As income levels increased by country, there has been clear increasing trends of the prevalence of raised fasting blood glucose and total cholesterol, overweight/obesity, and deaths from cancers. Alternatively, as national income declined, the prevalence of raised blood pressure and NCDs deaths, mortality from CVDs, diabetes, and chronic respiratory diseases tended to increase. In terms of diet factors, clear increasing trends in total energy, fat, and protein intake, and animal fat, vegetable oil, milk, stimulants, and sugar and sweetener supplies were observed with the increase in income-level countries, whereas increasing trends in carbohydrates intake, cereal supply, and alcohol consumption were observed with the decrease in income-level countries. Additionally, the same geographical pattern was observed for some factors. The share of raised blood pressure and deaths from NCDs and CVDs was higher in the LLIMCs of Asia, Africa, Latin America, and Europe. Carbohydrate intake was higher, and protein and fat intake were lower in low-income Asian and African countries than high-income European countries.

This study is the first to examine NCDs prevalence and all major behavioral risk factors across income levels and global regions. Our findings substantially augment the scant evidence from previous LLMIC-based studies on the prevalence of NCDs; 80% of NCDs deaths were found in LLMICs, of which almost 30% have a population under 60 years of age, and Africa and other LLMICs were predicted to have a rise in NCDs mortality in 2020 [19]. We found significantly wide differences in metabolic risk factors across national income levels with the differences in NCDs deaths, including cancers, CVDs, diabetes, and chronic respiratory diseases. Blood pressure was decreasing in HICs and some middle-income regions. In contrast, it was unchanged or increased in several LICs [20], and our results also showed that raised blood pressure was highest in LICs, which is the leading metabolic risk factor globally [21].

Additionally, we found that LLMICs had a higher mortality from CVDs, diabetes, and chronic respiratory diseases than did HICs, whereas cancer mortality was lower. A previous review demonstrated that the mortality has shifted from HICs to LLMICs because metabolic risk factors such as blood pressure, cholesterol, and CVDs rates have decreased in HICs. In contrast, the risk factors have increased or remained in LLMICs [22], consistent with our results except for cholesterol. Obesity is becoming increasingly common in LMICs [23], but our results showed that obesity is a decreasing trend with decreasing income level. A previous study showed that all HICs had a higher obesity prevalence than undernutrition, but LLMICs had a higher undernutrition prevalence than obesity [21]. Thus, substantial resources should be assigned for NCDs in LLMICs with very low resources and control undernutrition [24].

An unhealthy diet causes a greater NCDs burden than other health-related behaviors do [25]. The WHO reported that poor diet is one of the major factors affecting the development of NCDs risk factors such as blood pressure, glucose, lipids, and BMI [4]. The diet is found in both high- and low-income countries [26]. To improve this, it is necessary to develop policies suitable for situating each country [25]. Our risk factor findings mirror the established inequalities in the world. We found that HICs have the highest total energy, fat, and protein intake, and meat, animal fat, stimulants, and sugar and sweetener supplies, which are lowest in LLMICs. Carbohydrate intake along with cereal supply was higher in LLMICs with higher raised blood pressure and NCDs deaths. Numerous epidemiological studies have shown the positive or negative effects on specific foods or nutrients such as fats and oil, salt, sugar-sweetened beverages (SSB), and fruits and vegetables [27,28]. In HICs, fat and SSB may contribute to overweight/obesity and raised total cholesterol [27]. In LICs, 81.6% of people consume fewer vegetables than recommended by the WHO [29], which could contribute to micro-nutrient deficiency. Thus, the double burden of micro-nutrient deficiency and unhealthy diets should be focused on in LLMICs [27].

Regarding health-related behavioral factors, we found a tendency to increase insufficient physical activity as income levels increased. This is consistent with previous studies that insufficient physical activity was higher in upper-middle-income countries than in lower-middle-income countries [8]. The association between insufficient physical activity and obesity is well-known [8,30]. It can support our findings that obesity tends to increase with income level. In addition, we confirmed a tendency to increase alcohol consumption as income levels decrease. Previous studies have shown that low- and middle-income countries have higher rates of increases in alcohol consumption per capita than high-income countries do [31]. With the aim of reducing the prevalence of insufficient physical activity and harmful use of alcohol by at least 10% in the country, the WHO proposed the reduction in the NCDs burden [32].

Regional differences within a similar income level are essential to consider the global double burden of diseases [21]. Similar to our results, the previous review showed that the highest CVDs were shown in Eastern Europe and Central Asia, and CVDs were high in South Asia, North Africa, and the Middle East [33]. CVDs are the leading cause of death in every region of the world except for sub-Saharan Africa, where infectious diseases are the leading cause of mortality, and South Korea and Japan, where cancers are the main deaths [33]. The trend of dietary factors was heterogeneous by the world regions. Dietary patterns in HICs were improved, but those in several LICs (e.g., Africa and Asia) were worsened [27], where carbohydrate intake was higher, but protein intake was lower than HICs in our results. There are disparities in the dietary consumption by socioeconomic status within some countries [23,34]. For the fat intake, South and East Asia, South America, and certain Caribbean nations have the lowest intake [15], consistent with our results.

This study has several limitations. First, this study did not consider the cultural aspects, ethnicity, health systems, and availability of medical treatment of countries and other factors. Culture is one of the main factors that determine food behavior. However, in this study, culture was not considered. The reason was that it is difficult to express various cultures as united values. Ethnicity is related to the prevalence of NCDs and food culture. However, the relationship between race and NCDs was not fully discovered. There were various standards for asserting ethnicities. In addition, across countries, the variety and composition of races were diverse, making it difficult to represent them as a numeral or categorical variable.

The health system and availability of medical treatment affect the prevalence and deaths from NCDs. For example, low-income countries show a lower awareness, treatment, and control of hypertension [35]. However, this study did not consider the health system and availability of medical treatment. Second, various lifestyle factors were not considered as risk factors. NCDs are associated not only with inappropriate dietary habits but also with several inappropriate lifestyle habits, including smoking, alcohol consumption, physical activity, fast food consumption, skipping meals, and inappropriate sleeping [36]. However, there were no lifestyle-related factors in the data we used except smoking, alcohol consumption, and physical activity. The lifestyle factors were so diverse and broad that it was difficult to use them all, and only the main factors suggested by the WHO were included [12]. Another limitation of this study is that food supply was used to determine the food intake of each country. Among other data, food supply from the FAO had the highest number of nations where the data were collected. As food supply indicates food commodities in terms of production, imports, and stock changes, food supply is not an accurate indicator of the daily intake of foods. In addition, as the overall diet composition is unknown due to the absence of a diet survey, the dietary pattern cannot be analyzed.

Despite this, the present study has the following strengths. First, previous studies covered the effects of one or few food groups by income level [29]. This study investigated nine food groups or nutritional compositions and found different patterns in NCDs and income levels between the food groups. In addition, previous research covered trends of one type of NCDs [35]. In this study, five metabolic risk factors and four mortality rates of NCDs were discovered; each of these factors showed a different trend. This study investigated interactive trends of various kinds of NCDs-related factors and food groups in an integrative way. In addition, these trends were analyzed for countries of four income levels. This study was the first to investigate the relationships between diet and NCDs and to establish the difference between income levels of countries. Trends of NCDs and diets can be applied as basal data for international health policies fitted to each income-level country. This viewpoint is essential to determine appropriate policies for countries of different statuses. Another strength is that this study used GIS. By using GIS, the results were expressed as visualized figures. It is a helpful way to adopt various kinds of information using an integral perspective [9]. Figures of GIS maps in this study could be used to make the public better understand the trend of NCDs.

Following these findings, the direction of nutritional policy could be different from income levels. For HICs that experienced rapid declines in NCDs mortality, reductions in sugar and sweetener, as well as animal fat consumption, should be emphasized more than other food groups in policymaking. The policy might include guidelines and education on reducing diet for sugar and sweetener and animal fat. By contrast, in LLIMCs, the reduction in the prevalence of hypertension, a halt in the rise in undernutrition, and the reduction in alcohol consuming should be emphasized. Thus, vegetable consumption should be encouraged through national support, including education. Considering these findings, materials for education can be produced. In addition, policymakers should focus more on faster nutrition transition in the high-SES group in low-income countries related to NCDs trends [37]. By reflecting nutrition transition and NCDs trends, other SES groups need education and medical care for appropriate diets to prevent increasing NCDs. However, low-income countries lack health-related infrastructures for preventing NCDs. These findings could emphasize the importance of health-related infrastructures. International institutions or nongovernmental organizations should consider these findings when supporting various income levels of countries to promote global health.

In conclusion, in this study, different diets and NCDs trends were found across countries of different income levels, and raised blood pressure and dietary factors such as higher carbohydrates and alcohol consumption, a lower consumption of vegetables, vegetable oil, and milk are immediate health threats in lower-middle-income and low-income countries. On the other hand, a high intake of total energy, fat, and protein tended to increase total cholesterol and overweight/obese in high-income countries. Therefore, appropriate health policies for each group of countries are needed to solve the increasing challenges of NCDs.

## Figures and Tables

**Table 1:** Characteristics of the metabolic risk factors of NCDs and mortality of NCDs by income level ^1^.

		Income Levels (*n* = 151)				
		High Income (*n* = 39)	Upper-Middle Income (*n* = 43)	Lower-Middle Income (*n* = 38)	Low Income (*n* = 31)	*p* for Trend ^2^
**Prevalence**						
Raised fasting blood glucose ^3^	%	8.2 ± 2.2	10.0 ± 1.7	9.5 ± 2.1	7.7 ± 1.1	<0.001
Raised total cholesterol ^4^	%	16.3 ± 3.7	9.1 ± 2.8	6.0 ± 1.8	4.1 ± 0.7	<0.001
Overweight ^5^	%	57.6 ± 12.8	43.2 ± 12.2	26.7 ± 10.4	22.1 ± 4.3	<0.001
Obesity ^6^	%	24.1 ± 9.2	13.3 ± 8.5	7.3 ± 5.5	5.3 ± 2.1	<0.001
Raised blood pressure ^7^	%	18.9 ± 5.4	20.8 ± 3.2	25.6 ± 2.1	29.7 ± 2.9	<0.001
**Deaths** ^8^						
Risk of premature death from target NCDs ^9^	%	14.6 ± 6.1	18.9 ± 3.1	23.6 ± 3.5	20.3 ± 4.5	<0.001
NCDs deaths under age 70 ^10^	% (Male)	36.6 ± 9.7	44.3 ± 6.8	60.4 ± 7.3	65.6 ± 6.3	<0.001
NCDs deaths under age 70	% (Female)	22.0 ± 8.1	35.7 ± 6.6	51.6 ± 9.8	60 ± 8.2	<0.001
**Deaths (per 100,000)**						
All NCDs ^11^	Numbers	416 ± 149	564 ± 81	663 ± 73	636 ± 124	<0.001
Cancers	Numbers	122 ± 17	127 ± 23	88 ± 19	104 ± 29	<0.001
Cardiovascular diseases	Numbers	178 ± 134	285 ± 73	306 ± 74	277 ± 97	<0.001
Diabetes	Numbers	11 ± 8	25 ± 22	35 ± 16	36 ± 13	<0.001
Chronic respiratory diseases	Numbers	24 ± 8	60 ± 20	101 ± 52	57 ± 33	<0.001

^1^ Data were weighted by total population. ^2^ P for trends were calculated by variance-weighted least-squares regression analysis. ^3^ Data were age-standardized. Subjects were adults older than 25 years old who have raised fasting blood glucose ≥ 7.0 mmol/L or are on medication (2014). ^4^ Data were age-standardized. Subjects who were older than 25 years old who have raised total cholesterol ≥ 6.2 mmol/L (2008). ^5^ Data were age-standardized. Subjects were adults older than 18 years old with body mass index (kg/m^2^) ≥ 25 (2014). ^6^ Data were age-standardized. Subjects were adults older than 18 years old with body mass index (kg/m^2^) ≥ 30 (2014). ^7^ Data were age-standardized. Subjects were adults older than 18 years old who have systolic blood pressure over 140 mmHg or diastolic blood pressure over 90 mmHg (2014). Source: Data were from http://apps.who.int/gho/data/ (accessed on 11 October 2021). ^8^ Counted numbers of deaths (2012). ^9^ Risk of premature death from target NCDs presents the probability of dying between exact ages of 30 and 70 from any CVDs, cancers, diabetes, or chronic respiratory disease (2012). ^10^ Data were weighted by total population. Deaths under age 70 are a percent of all NCDs deaths (2012). ^11^ The numbers of deaths per 100,000 population were adjusted for differences in the age distribution of the population by applying the observed age-specific mortality rates for each population to a standard population (2012). P for trends was calculated by variance-weighted least-squares regression analysis.

**Table 2:** Nutrition compositions, food supply, and health-related behaviors according to income level, including total energy intake, fat content, and protein content ^1,2^.

	Income Levels				
	High Income	Upper-Middle Income	Lower-Middle Income	Low Income	*p* for Trend ^3^
**Nutrition consumption**					
Total energy intake (kcal/capita/day)	3355 ± 287	3067 ± 206	2561 ± 254	2274 ± 210	<0.001
Fat (g/capita/day)	132.9 ± 28.4	92.6 ± 14.2	55.7 ± 11.0	42.8 ± 12.8	<0.001
Fat (%) ^4^	35.4 ± 5.7	27.1 ± 3.2	19.6 ± 3.6	16.7 ± 4.2	<0.001
Protein (g/capita/day)	101.8 ± 9.8	90.8 ± 10.6	64.3 ± 10.0	59.4 ± 9.4	<0.001
Protein (%) ^5^	12.1 ± 0.7	11.8 ± 0.9	10.0 ± 1.0	10.5 ± 1.3	<0.001
Carbohydrates (%) ^6^	52.5 ± 5.8	61.1 ± 3.6	70.4 ± 4.3	72.8 ± 4.0	<0.001
**Food supply**					
Fruit	108.2 ± 35.2	108.4 ± 38.7	80.3 ± 41.9	69.5 ± 83.2	<0.001
Vegetable	80.0 ± 24.7	151.3 ± 82.1	54.8 ± 24.8	30.4 ± 23.1	<0.001
Cereal	934.5 ± 197	1329.9 ± 211.4	1406.5 ± 235.7	1352.8 ± 390.5	<0.001
Meat	370.5 ± 89.5	369.9 ± 120.3	69 ± 90.8	68.6 ± 52.8	<0.001
Animal fat	134.6 ± 85.8	48 ± 24.3	53 ± 30.8	15.4 ± 9.8	<0.001
Vegetable oil	538.8 ± 154.2	262.7 ± 102.8	216.7 ± 59.2	156.5 ± 71.3	<0.001
Milk	286.7 ± 106.7	122.8 ± 95.4	109.7 ± 80.5	62.5 ± 57.2	<0.001
Stimulant	24.8 ± 12.2	5.7 ± 6.8	2.9 ± 4.4	2.4 ± 4	<0.001
Sugar and sweetener	415.4 ± 119.4	213.1 ± 167	208 ± 74.2	98.7 ± 48.7	<0.001
**Health-related behaviors**					
Smoking ^7^	32.1 ± 12.8	41.3 ± 11.7	33.1 ± 16.7	23.5 ± 11.9	<0.001
Insufficient activity ^8^	28.2 ± 10.1	27.3 ± 8.4	18.3 ± 6.1	14.4 ± 6.9	<0.001
Alcohol consumers, past 12 months ^9^	38.5 ± 27.8	36.3 ± 13.7	25.3 ± 29.3	41.0 ± 23.6	<0.001

^1^ Food supplies are derived from the total supplies available for human consumption by dividing the quantities of food by the total population actually partaking of the food supplies during the reference period within the present geographical boundaries of the country. ^2^ Data were weighted by total population. ^3^ P for trends was calculated by variance-weighted least-squares regression analysis. ^4^ Percentage of fat consumption within the total calorie intake. ^5^ Percentage of protein consumption within the total calorie intake. ^6^ Percentage of carbohydrates consumption within the total calorie intake. Source: Data were from http://fenix.fao.org/faostat/beta/en/ (accessed on 11 October 2021). ^7^ Data were weighted by total population. Data were age-standardized. Subjects were adults who are older than 15 years old and smoke. Data were collected from analysis of the full set of adult tobacco use surveys (or surveys that ask tobacco-use questions). Tobacco includes cigarettes, cigars, pipes, or any other smoked tobacco products. Current smoking describes both daily and nondaily or occasional smoking (2013). The following 41 countries were excluded from the smoking dataset due to the absence of data: Afghanistan, Algeria, Angola, Austria, the Bahamas, Belize, Bolivia (Plurinational State of), Botswana, Côte d’Ivoire, Central African Republic, Chad, Cyprus, the Democratic People’s Republic of Korea, Djibouti, El Salvador, Gabon, Gambia, Guatemala, Guinea, Guinea-Bissau, Guyana, Iraq, Kuwait, Madagascar, the Maldives, Nicaragua, Peru, Rwanda, the Solomon Islands, Sudan, Suriname, Tajikistan, The former Yugoslav republic of Macedonia, Togo, Trinidad and Tobago, Tunisia, Turkmenistan, the United Arab Emirates, Venezuela (the Bolivarian Republic of), Yemen, and Zimbabwe. ^8^ Data were age-standardized. Subjects were adults older than 18 years old who attain less than 150 min of moderate-intensity physical activity per week, or less than 75 min of vigorous-intensity physical activity per week or equivalent. Data were based on self-reported physical activity captured using the GPAQ (Global Physical Activity Questionnaire), the IPAQ (International Physical Activity Questionnaire), or a similar questionnaire covering activity at work/in the household, for transport, and during leisure time (2010). The following 33 countries were excluded from insufficient-activity dataset due to the absence of data: Afghanistan, Albania, Angola, Armenia, Azerbaijan, Belarus, Belize, Bolivia (Plurinational State of), Brunei Darussalam, Costa Rica, Cuba, the Democratic People’s Republic of Korea, Djibouti, El Salvador, Guinea-Bissau, Guyana, Haiti, Honduras, Iceland, Israel, Morocco, Nicaragua, Panama, Peru, Sudan, Suriname, Switzerland, Tajikistan, The former Yugoslav republic of Macedonia, Turkmenistan, Uganda, Venezuela (the Bolivarian Republic of), and Yemen. ^9^ Alcohol consumers in the past 12 months are defined as the proportion of adults (15+ years) in a given population who have consumed any alcohol during the past 12 months, assessed at a given point in time (2010). Source: Data were from http://apps.who.int/gho/data/ (accessed on 11 October 2021).

## References

[1] Renzella J., Townsend N., Jewell J., Breda J., Roberts N., Rayner M., Wickramasinghe K. (2018). What National and Subnational Interventions and Policies Based on Mediterranean and Nordic Diets Are Recommended or Implemented in the WHO European Region, and Is There Evidence of Effectiveness in Reducing Noncommunicable Diseases?.

[2] Sustainabledevelopment.un.org (2019). The Future Is Now-Science for Achieving Sustainable Development. https://sustainabledevelopment.un.org/gsdr2019.

[3] World Health Organization (2018). Noncommunicable Diseases Country Profiles. https://apps.who.int/iris/handle/10665/274512.

[4] World Health Organization (2014). Global Status Report on Noncommunicable Diseases. https://apps.who.int/iris/handle/10665/148114.

[5] (2011). World Health Organization Scaling up Action against NCDs: How Much Will It Cost.

[6] Clark H. (2013). NCDs: A challenge to sustainable human development. Lancet.

[7] Roth G.A., Huffman M.D., Moran A.E., Feigin V., Mensah G.A., Naghavi M., Murray C.J. (2015). Global and regional patterns in cardiovascular mortality from 1990 to 2013. Circulation.

[8] Wu F., Guo Y., Chatterji S., Zheng Y., Naidoo N., Jiang Y., Biritwum R., Yawson A., Minicuci N., Salinas-Rodriguez A. (2015). Common risk factors for chronic non-communicable diseases among older adults in China, Ghana, Mexico, India, Russia and South Africa: The study on global AGEing and adult health (SAGE) wave 1. BMC Public Health.

[9] Brown G. (2004). Mapping spatial attributes in survey research for natural resource management: Methods and applications. Soc. Nat. Resour..

[10] World Health Organization. https://www.who.int/data/gho/indicator-metadata-registry/imr-details/523.

[11] World Bank. https://data.worldbank.org/.

[12] (2014). World Health Organization, Global Health Statistics. http://apps.who.int/gho/data/.

[13] Singh G.M., Micha R., Khatibzadeh S., Shi P., Lim S., Andrews K.G., Engell R.E., Ezzati M., Mozaffarian D. (2015). Global Burden of Diseases Nutrition and Chronic Diseases Expert Group (NutriCoDE) Global, regional, and national consumption of sugar-sweetened beverages, fruit juices, and milk: A systematic assessment of beverage intake in 187 countries. PLoS ONE.

[14] Wang X., Ouyang Y., Liu J., Zhu M., Zhao G., Bao W., Hu F.B. (2014). Fruit and vegetable consumption and mortality from all causes, cardiovascular disease, and cancer: Systematic review and dose-response meta-analysis of prospective cohort studies. BMJ.

[15] Micha R., Khatibzadeh S., Shi P., Fahimi S., Lim S., Andrews K.G., Engell R.E., Powles J., Ezzati M., Mozaffarian D. (2014). Global, regional, and national consumption levels of dietary fats and oils in 1990 and 2010: A systematic analysis including 266 country-specific nutrition surveys. BMJ.

[16] Stevens G.A., Singh G.M., Lu Y., Danaei G., Lin J.K., Finucane M.M., Bahalim A.N., McIntire R.K., Gutierrez H.R., Cowan M. (2012). National, regional, and global trends in adult overweight and obesity prevalences. Popul. Health Metr..

[17] (2017). The WHO Recommendations for the Diagnostic Criteria. http://apps.who.int/gho/data/.

[18] Food and Agriculture Organization of the United Nations. http://www.fao.org/home/en/.

[19] (2008). World Health Organization The Global Burden of Disease: 2004 Update. https://apps.who.int/iris/handle/10665/43942.

[20] Danaei G., Finucane M.M., Lin J.K., Singh G.M., Paciorek C.J., Cowan M.J., Farzadfar F., Stevens G.A., Lim S.S., Riley L.M. (2011). National, regional, and global trends in systolic blood pressure since 1980: Systematic analysis of health examination surveys and epidemiological studies with 786 country-years and 5· 4 million participants. Lancet.

[21] Min J., Zhao Y., Slivka L., Wang Y. (2018). Double burden of diseases worldwide: Coexistence of undernutrition and overnutrition-related non-communicable chronic diseases. Obes. Rev..

[22] Danaei G., Lu Y., Singh G.M., Carnahan E., Stevens G.A., Cowan M.J., Farzadfar F., Lin J.K., Finucane M.M., Rao M. (2014). Cardiovascular disease, chronic kidney disease, and diabetes mortality burden of cardiometabolic risk factors from 1980 to 2010: A comparative risk assessment. Lancet Diabetes Endocrinol..

[23] Niessen L.W., Mohan D., Akuoku J.K., Mirelman A.J., Ahmed S., Koehlmoos T.P., Trujillo A., Khan J., Peters D.H. (2018). Tackling socioeconomic inequalities and non-communicable diseases in low-income and middle-income countries under the Sustainable Development agenda. Lancet.

[24] Stringhini S., Bovet P. (2017). Socioeconomic status and risk factors for non-communicable diseases in low-income and lower-middle-income countries. Lancet Glob. Health.

[25] Hyseni L., Atkinson M., Bromley H., Orton L., Lloyd-Williams F., McGill R., Capewell S. (2017). The effects of policy actions to improve population dietary patterns and prevent diet-related non-communicable diseases: Scoping review. Eur. J. Clin. Nutr..

[26] Hayashi F., Takemi Y. (2015). Why Is Creating a Healthy Food Environment So Crucial to Making Improvements in Diet-Related NCDs?. J. Nutr. Sci. Vitaminol..

[27] Ronto R., Wu J.H., Singh G.M. (2018). The global nutrition transition: Trends, disease burdens and policy interventions. Public Health Nutr..

[28] Afshin A., Sur P.J., Fay K.A., Cornaby L., Ferrara G., Salama J.S., Mullany E.C., Abate K.H., Abbafati C., Abebe Z. (2019). Health effects of dietary risks in 195 countries, 1990–2017: A systematic analysis for the Global Burden of Disease Study 2017. Lancet.

[29] Hall J.N., Moore S., Harper S.B., Lynch J.W. (2009). Global variability in fruit and vegetable consumption. Am. J. Prev. Med..

[30] Fonseca-Junior S.J., Sá C.G., Rodrigues P.A., Fernandes-Filho A.J. (2013). Physical exercise and morbid obesity: A systematic review. Arq. Bras. Cir. Dig..

[31] Moodie R., Stuckler D., Monteiro C., Sheron N., Neal B., Thamarangsi T., Lincoln P., Casswell S. (2013). Lancet NCD Action Group. Profits and pandemics: Prevention of harmful effects of tobacco, alcohol, and ultra-processed food and drink industries. Lancet.

[32] World Health Organization (2014). Noncommunicable Diseases Global Monitoring Framework: Indicator Definitions and Specifications. https://www.who.int/publications/i/item/ncd-gmf-indicator-definitions-and-specifications.

[33] Benziger C.P., Roth G.A., Moran A.E. (2016). The global burden of disease study and the preventable burden of NCD. Glob. Heart.

[34] Estimé M.S., Lutz B., Strobel F. (2014). Trade as a structural driver of dietary risk factors for noncommunicable diseases in the Pacific: An analysis of household income and expenditure survey data. Glob. Health.

[35] Finucane M.M., Stevens G.A., Cowan M.J., Danaei G., Lin J.K., Paciorek C.J., Singh G.M., Gutierrez H.R., Lu Y., Bahalim A.N. (2011). National, regional, and global trends in body-mass index since 1980: Systematic analysis of health examination surveys and epidemiological studies with 960 country-years and 9· 1 million participants. Lancet.

[36] Habib A., Alam M.M., Hussain I., Nasir N., Almuthebi M. (2020). Erratic Behavioral Attitude Leads to Noncommunicable Diseases: A Cross-Sectional Study. Biomed Res. Int..

[37] Popkin B.M. (2004). The nutrition transition: An overview of world patterns of change. Nutr. Rev..

